# Early Lifestyle Intervention for Obesity Prevention in Pediatric Survivors of Acute Lymphoblastic Leukemia

**DOI:** 10.3390/nu11112631

**Published:** 2019-11-02

**Authors:** Fang Fang Zhang, Michael Kelly, Mengxi Du, Jennifer J. G. Welch, Nadine Santacruz, Jacqueline Rhoades, Christina Luongo Kamins, ZoAnn Dreyer, Michael E. Scheurer

**Affiliations:** 1 Friedman School of Nutrition Science and Policy, Tufts University, 150 Harrison Ave, Boston, MA 02111, USA; mengxi.du@tufts.edu; 2 The Floating Hospital for Children, Tufts Medical Center, Boston, MA 02111, USA; michaelkelly128@gmail.com; 3 Hasbro Children’s Hospital, Brown University, Providence, RI 02903, USA; jwelch@lifespan.org; 4 Eastern Maine Medical Center, Brewer, ME 04412, USA; nsantacruz@emhs.org; 5 Driscoll Children’s Hospital, Corpus Christi, TX 78411, USA; Jacqueline.Rhoades@dchstx.org; 6 Independent Researcher, West Chester, PA 19380, USA; christinal79@yahoo.com; 7 Cancer and Hematology Centers, Texas Children’s Hospital, Houston, TX 77030, USA; zedreyer@txch.org; 8 Department of Pediatrics, Baylor College of Medicine, Houston, TX 77030, USA; scheurer@bcm.edu

**Keywords:** lifestyle, intervention, childhood cancer, nutrition, physical activity

## Abstract

Patients with pediatric acute lymphoblastic leukemia (ALL) experience rapid weight gain during treatment and increases in weight are maintained throughout treatment and beyond. Without prompt interventions, altered dietary and physical activity behaviors may become difficult to reverse, contributing to obesity risk long-term. Fifteen children, aged 3–9 years, diagnosed with pediatric ALL who were on maintenance therapy or within two years of treatment completion (mean BMI percentile: 70.4^th^) and one parent from each family, were enrolled into a 12-week lifestyle intervention delivered remotely through web-based sessions and phone calls with a lifestyle coach. Outcomes were assessed at baseline and end of the intervention. Thirteen of the 15 enrolled families (86.7%) completed the intervention. Parents reduced the “pressure to eat” feeding practice (change in mean score: −0.60, 95% CI: −1.12 to −0.07; *p*-value = 0.03) post intervention. Children increased the consumption of milk (0.54 serving/d, 0.02 to 1.07; *p*-value = 0.04) and percent of calories from protein (2.54%, 0.22 to 4.87%; *p*-value = 0.04) and reduced the consumption of potatoes (−0.16 serving/d, -0.30 to −0.03; *p*-value = 0.02). No significant changes were observed for children’s levels of physical activity, BMI, or waist circumference. Results from this pilot support the feasibility and preliminary efficacy of early lifestyle intervention among pediatric ALL survivors.

## 1. Introduction

Acute lymphoblastic leukemia (ALL) is the most common type of cancer diagnosed in children. Pediatric patients with ALL are not only are at a high risk of obesity [1] but also experience unhealthy weight gain early in treatment [2,3,4,5,6]. Prior studies reported rapid weight gain during induction and early phases of maintenance therapy, and increases in weight are maintained throughout treatment and beyond [2,3,4,5,6]. Furthermore, cardiovascular disease (CVD) risk factors such as insulin resistance and hypertension occur early during treatment for pediatric ALL patients [4,7]. When patients become long-term survivors, they develop severe, life threatening, or fatal conditions more frequently and at a much younger age than their siblings [8,9]. It is possible that early onset of obesity and obesity-related morbidities contribute to a high chronic disease burden among long-term survivors of childhood cancer. These findings provide strong support to integrate lifestyle interventions early in cancer care to address the early onset of obesity and CVD risk factors among pediatric ALL patients. 

Weight gain occurs when levels of energy intake exceed levels of energy expenditure. Existing studies provide consistent evidence that childhood cancer survivors have poor dietary intake patterns such as low consumption of whole grains and dietary fiber and high consumption of sodium and empty calories (calories from solid fats and added sugars) [10,11,12,13,14,15]. Childhood cancer survivors also have low levels of physical activity [16,17,18,19]. Both are well-established risk factors for obesity and CVD risk. Although the treatments for childhood cancer put them at a high risk for obesity and cardiometabolic conditions [20], psychosocial factors may also play important roles in affecting dietary intake patterns and physical activity levels of young patients diagnosed with childhood cancer. For example, prior studies found that parents became more permissive in parenting styles after their child is diagnosed with cancer, allowing the child to eat whatever he or she can or wants and encouraging sedentary behavior [21]. These changes in parenting style and practices, although originally thought to be acute responses to the child’s cancer diagnosis and treatment, are often carried over long into survivorship [7,22,23]. Without prompt interventions, altered dietary and physical activity behaviors may become difficult to reverse when pediatric cancer patients become long-term survivors. 

We reported here the feasibility and initial efficacy of an early lifestyle intervention on enhancing parenting style and practices, improving dietary intake and levels of physical activity, and preventing excess weight gain among patients with pediatric ALL who were on treatment or within two years of treatment completion. 

## 2. Materials and Methods 

### 2.1. Study Population

Between 2014 and 2016, patients diagnosed with pediatric ALL and one parent from each family were recruited from patients attending the three oncology clinics in New England (Floating Hospital for Children in Boston, Massachusetts; Eastern Maine Medical Center in Bangor, Maine; and Hasbro Children’s Hospital in Providence, Rhode Island) and two oncology clinics in Texas (Texas Children’s Hospital in Houston and Driscoll Children’s Hospital in Corpus Christi). Eligible families had children who (1) were diagnosed with ALL, (2) were on maintenance chemotherapy or within 2 years post-treatment, (3) were 3–11 years at enrollment, and (4) parents who had regular access to internet or mobile phones. Children were excluded if they (1) had recurrent cancer, (2) had Down syndrome or intellectual disability, (3) were allogeneic transplant recipients, (4) were participating in other lifestyle intervention programs, or (5) had chronic conditions affecting energy balance. Informed consent was obtained from parents and assent was obtained from children aged 10 years or older before they participated in the study. Following consent, parents completed self-administered questionnaires that asked about demographics, parenting style and practices. Information on child’s cancer diagnosis and treatment such as age at diagnosis and exposure to cancer treatment were extracted from medical records using the Summary of Cancer Treatment form by the Children’s Oncology Group [24]. Eligible families meeting the inclusion criteria were invited to participate in the intervention. The study was conducted with the Declaration of Helsinki, and the protocol was approved by the Institutional Review Board (IRB) at each participating institution (Tufts Health Science Campus IRB #11581).

### 2.2. Intervention

The design of the Healthy Eating and Active Living (HEAL) intervention has been described elsewhere [25]. Briefly, the HEAL program consisted of 12 weekly self-guided educational and behavioral sessions available online with printed hard copies made available to participants per requests. The initial curriculum was developed based on the “4-Health” program, a childhood obesity prevention program in rural settings by the US Department of Agriculture (USDA) [26]. The adaption of the curriculum was directly informed by qualitative research that identified targeted nutrition and physical activity needs and specific barriers for childhood ALL survivors [27]. Grounded in theoretical models (e.g., social cognitive and self-determination theories) [28,29], the intervention sessions incorporated core behavioral change strategies including increasing knowledge, setting behavior goals, self-monitoring, problem solving, and positive reinforcement [30]. Enrolled families were scheduled for an initial phone call with a lifestyle coach who provided an overview of the program goals and introduced program materials, followed by weekly sessions through a web-based program outlining positive parenting style and practices, healthy eating, and physical activity for 12 weeks.

The intervention had three main focuses on behavioral change: (1) Positive parenting style and practices—The intervention engaged parents as the “agent of change” and emphasized parents’ roles in transitioning the family towards healthy eating and active living through modifying the family environment. The intervention included sessions for positive parenting practices to facilitate child’s healthy eating and physical activity. (2) Healthy eating—The intervention promoted an overall healthy eating pattern by limiting consumption of sugar-sweetened beverages (SSBs) and snacks high in empty calories; limiting consumption of processed foods and snacks high in sodium; increasing consumption of vegetables, fruits, and whole grains, and increasing dietary source of vitamin D and calcium from both dairy and non-dairy products. (3) Physical activity—The intervention also aimed to avoid inactivity and increase activity by reducing screen time, gradually increasing activity to ≥60 min/day and incorporating physical activity into daily activities, and incorporating bone-strengthening activity on ≥3 day/week [31,32].

Specific sessions were incorporated to address barriers experienced by survivors and families for making healthy food choices or being physical active, such as overcoming food cravings, coping with changes in taste preference and fatigue, curbing emotional eating, safety concerns with physical activity, and stress and time management. To facilitate positive parenting practices in nutrition and physical activity, parents were asked to set SMART goals throughout the program that resonated with their family needs. These goals were to be “Specific”, “Measurable”, “Achievable”, “Relevant”, and “Time-bound” [33]. Parents also completed weekly food and activity logs to track eating and activity behaviors for the child. A lifestyle coach monitored the logs, checked in on SMART goals, and provided feedback with conversations guided by motivational interviewing during weekly phone calls to encourage and sustain behavioral changes. 

### 2.3. Outcomes

#### 2.3.1. Dietary Intake

Dietary intake data for 24 hour recalls were collected and analyzed using the Automated Self-Administered 24 hour (ASA24) Dietary Assessment Tool developed by the National Cancer Institute [34,35]. A set of three 24 hour diet recalls were collected, including two weekdays and one weekend, were administered with parents within two weeks of the baseline/follow-up visit. Our prior study in childhood cancer survivor demonstrated that repeated 24 hour diet recalls provided valid estimates for dietary intake in reference to the doubly labeled water method [36].

#### 2.3.2. Physical Activity

Physical activity was assessed using accelerometers. Children were asked to wear an Actigraph GT1M Monitor over the right hip on an elasticized belt for seven consecutive days at baseline and follow-up visits. They were instructed to wear the device while they are awake and take it off for swimming or bathing. Accelerometers have been used to collect objective data on physical activity for youth 3–15 years in NHANES [37] and other studies [38]. We adopted the same criteria used in NHANES to define a valid day and minutes per week spent in moderate and vigorous physical activity according to count threshold [39].

#### 2.3.3. Anthropometrics

Child’s weight (±0.1 kg) was measured in the clinic on a calibrated digital scale in light clothing without shoes, and height (±0.1 cm) was measured using a wall mounted stadiometer. Child’s waist circumference (±0.1 cm) was measured by placing measurement tapes around the abdomen. Standard protocols were used for anthropometrics at each clinic. 

#### 2.3.4. Parenting Style and Practices

Parenting style was assessed using the Parenting Dimensions Inventory Short Version (PDI-S). PDI-S is a parent-administered instrument that examines five dimensions of parenting (nurturance, type of control, amount of control, inconsistency, and family organization) [40,41], and allows the categorization of parenting styles into four types: authoritative, authoritarian, permissive, and uninvolved [42]. Parenting practices were measured by items adapted from the Child Feeding Questionnaire (CFQ) [43] that measure three feeding practice subscales (restriction, monitoring, and pressure to eat) and items that measure logistical support, explicit modeling, and limit setting in relation to child’s physical activity [44,45].

### 2.4. Statistical Analyses 

We first assessed the participation, adherence, and retention rate of the intervention. Participation rate was determined by the percentage of parents who were approached for participation agreed to participate and were enrolled, adherence rate was determined by the percentage of enrolled participants who completed 80% or more of the scheduled sessions at 6 month follow-up, and retention rate was determined by the percentage of enrolled participants who were retained and completed the end of study assessments.

We then assessed changes in children’s dietary intake, levels of physical activity, and weight status and parents’ parenting style and practices before and after the intervention, using t-test for continuous outcomes and chi-square test for categorical outcomes. For weight status, BMI z-score and percentile were calculated using the 2000 Center for Disease Prevention and Control (CDC) growth charts [46]. For dietary intake patterns, multiple 24 hour diet recalls were averaged and presented on a density basis, counted as amount per 2000 kcal. The Healthy Eating Index (HEI)-2015 total scores and scores for 13 components were calculated to assess adherence to the 2015 Dietary Guidelines for Americans [47]. Levels of physical activity were assessed using minutes per day of light, moderate, and vigorous physical activities (MVPA). Parenting styles including authoritative style (high demandingness/high responsiveness), authoritarian style (high demandingness/low responsiveness), indulgent style (low demandingness/high responsiveness), and uninvolved style (low demandingness/low responsiveness) were determined based on the median scores of nurturance and amount of control subscales. Mean scores for parents’ feeding practices were assessed in three dimensions including pressure to eat, monitoring, and restriction, and for physical activity practices in three dimensions including logistic support, explicit modeling, and limit setting.

## 3. Results

Among the 30 families who consented, half (*n* = 15) enrolled into the study. During the intervention, one family dropped out after completing nine sessions due to child’s declining health; and one family was lost to follow-up after completing two sessions. Thirteen of the 15 enrolled families (86.7%) completed all 12 sessions and the end-of-study assessments. Among these 13 families, the average length of completing all 12 sessions was 13.7 weeks (range: 10.9 to 15.7 weeks), the mean time spent on reviewing each session online was 25 minutes (range: 11 to 32 minutes), and the mean time spent on weekly phone calls with the lifestyle coach was 30 minutes (range: 25 to 35 minutes). At study enrollment, the mean age of the 15 children was 6.1 years (standard deviation (SD): 2.0; range: 3.8–9.8) (Table 1), over two-thirds (73.3%) were boys, 60% were non-Hispanic whites, 13 (86.7%) were on maintenance therapy, and two were within two years of treatment completion. 

Among the 13 children who completed both the baseline and post-intervention assessments on outcomes, the consumption of milk increased by 0.54 (95% CI: 0.02 to 1.07) serving/d and the consumption of potatoes decreased by −0.16 (95% CI: −0.30 to −0.03) serving/d (Table 2). For nutrients, children increased dietary intake of calcium by 302 (95% CI: 25.1 to 579) mg/d and the percentage of calories from protein by 2.54% (95% CI: 0.22% to 4.87%). Children also decreased the consumption of added sugars by −14.6 (95% CI: −29.5 to 0.4) g/d and the difference was borderline significant. The overall diet quality measured by the HEI-2015 increased by 3.14 (95% CI: −3.55 to 9.82) but did not reach statistical significance. Parents reported reducing the “pressure to eat” feeding practice (mean change in mean score: −0.60, 95% CI: −1.12 to −0.07) (Table 3). The intervention did not result in significant changes in children’s weight status or levels of physical activity.

## 4. Discussion

In this parent-targeted early lifestyle intervention, pediatric ALL patients who were on maintenance therapy or within two years of treatment completion feasibly completed a 12 week program and demonstrated an increased dietary intake of milk, calcium, and protein, with some indications of improved carbohydrate quality. Parents reported reducing the “pressure to eat” feeding practice. The intervention did not lead to significant changes in children’s levels of physical activity or weight status. 

Family environment plays important roles in shaping children’s dietary and activity behaviors, [48,49,50,51,52,53] and parenting style and practices can be particularly important for children diagnosed with cancer at a young age. As reported in qualitative research, parents become more permissive or indulgent in parenting after child is diagnosed with cancer [21,54], and parents with the permissive or indulgent parenting style often have difficulties in setting appropriate limits in child’s eating and activity behaviors, or are unable to follow through with discipline when the child is upset [55]. Changes in parents’ feeding practices have also been previously reported in pediatric cancer patients [56]. For example, during the initial intensive phase of treatment, parents were encouraged to provide as many calories as they could to prevent their child’s weight loss and cope with treatment-associated side effects such as vomiting and nausea. Indeed, “pressure to eat” comes to be one of the major feeding practice themes reported by the parents during cancer treatment [56]. However, when excessive weight gain subsequently follows initial weight loss, parents often find difficulties in reversing unhealthy eating habits and sedentary behaviors that have been formed during the treatment [21]. 

To our best knowledge, our study was the first lifestyle intervention for childhood cancer survivors that incorporated changing parenting style and practices as a component for behavioral change. We did not find significant changes in parenting style, but parents reduced the “pressure to eat” feeding practice post intervention. It is possible that parenting style is less amenable to intervention than parenting practices, as suggested by prior studies [57]. In the general population, the “pressure to eat” feeding practice was associated with less consumption of healthy foods such as fruits and vegetables [58]. Thus, reduced “pressure to eat” in the feeding practice may have the potential to improve dietary intake patterns of pediatric ALL patients long-term. 

We found that an early lifestyle intervention significantly increased milk consumption and dietary intake of calcium among pediatric ALL patients. Before the intervention, the mean consumption of milk and calcium in pediatric ALL patients was comparable to that of the general pediatric population of similar age (6–9 years) in the National Health and Nutrition Examination Survey (NHANES) 2013–2014 (milk: 1.4 vs. 1.3 servings/d; calcium: 1070 vs. 1050 mg/d). After the intervention, pediatric ALL patients consumed 0.5 serving/d more milk and nearly 300 mg/d more calcium from dietary sources. This result is consistent with one previous intervention that aimed to improving bone health behaviors among adolescent survivors of childhood cancer: [59] nutrition counseling plus free provision of calcium supplements resulted in a significant increase in milk consumption and calcium intake from both food and supplement sources [59]. It has to be noted, however, additional calcium supplementation was not associated with improved bone mineral density in survivors of pediatric ALL [60]. These results, although reflecting short-term changes, suggest that eating a healthy and balanced diet that includes dairy products can potentially improve the bone mineral density among pediatric cancer patients. Our intervention also resulted in an increase in percent of calories from protein. The percent of calories from protein among pediatric ALL patients was lower than that in the general population before the intervention (12.4% vs. 14.4%) but reached 15.0% after the intervention. Such an increase could be partly attributed to an increased consumption of milk and having adequate intake of protein may help pediatric cancer patients maintain a healthy weight. 

In addition, we found that pediatric ALL survivors reduced consumption of potatoes along with a reduced consumption of added sugars, both of which are indicators for improved carbohydrate quality for better cardiometabolic health [61]. Before the intervention, the pediatric ALL survivors had similar consumption of white potatoes and added sugars to the general pediatric population (potatoes: 0.25 vs. 0.23 servings/d; added sugars: 68.0 vs. 65.6 g/d). After the intervention, the survivors reduced the consumption of white potatoes by 0.16 servings and added sugars by 15.3 grams per day. Change in HEI-2015 was in the same direction suggesting improvement in overall diet quality, although the difference did not reach statistical significance. Few prior lifestyle interventions among childhood cancer survivors included improving dietary intake patterns as a specific intervention component [62,63]. Our findings support the potential of early lifestyle interventions in improving dairy consumption, carbohydrate quality, and the overall diet quality among children with pediatric ALL. 

Increasing levels of physical activity, reducing sedentary behaviors, and improving physical functioning have been the focus of several lifestyle interventions in childhood cancer survivors. Increases in levels of physical activity were reported by some studies but not in others [64,65,66,67]. Similarly, no consistent findings on weight management were reported in previous studies. The inconsistent evidence of lifestyle interventions on childhood cancer survivors’ dietary intake patterns, levels of physical activity, and weight status may reflect the considerable heterogeneity in intervention design, duration, and outcome assessment. These interventions were usually small in size (<50 participants), the majority had a short duration (<6 months), and few conducted long-term assessments on children’s weight. For our intervention sessions, a larger focus was placed on improving dietary intake patterns than on increasing levels of physical activity. These factors, taken together, may have contributed to the lack of significant findings on changes in levels of physical activity and body weight in this pilot intervention. 

This study has several limitations. Due to the pilot nature of the intervention, a quasi-experiment design was used without controls and randomization, which does not facilitate causal relationship to be inferred from this study. Second, our sample size was small, which may have contributed to the lack of significant findings for the overall diet quality and other dietary components. Third, the shorter duration of the intervention and follow up may further limit our ability to identify significant changes in diet, levels of physical activity, and weight. Fourth, about half the eligible families did not participate in the study, which may further hinder the generalizability of our study findings to pediatric ALL patients as a whole. Because only patients with pediatric ALL were enrolled, the study findings may not be generalizable to other pediatric cancer patients. Fifth, assessing multiple dietary factors may lead to spurious findings due to multiple comparisons. Positive findings from this pilot study need to be interpreted with caution. 

Despite these limitations, our study is one of the few early lifestyle interventions with a specific focus on improving dietary intake patterns among pediatric ALL patients and additionally incorporated components for improving parenting style and practices to facilitate children’s lifestyle change. Our study findings demonstrated that early lifestyle intervention was feasible among pediatric ALL patients who are on treatment or shortly after treatment completion, which may capture the sensitive window of weight gain and behavioral adaptions. The initial efficacy revealed improvements in dietary intake patterns including increased consumption of milk, calcium, and protein and improved carbohydrate quality. These findings support the feasibility and preliminary efficacy of implementing a web- and phone-based lifestyle intervention early during the spectrum of survivorship. Further studies are required to evaluate whether early lifestyle intervention can lead to successful weight management long-term among pediatric ALL patients as well as other pediatric cancer patients and survivors. 

## Figures and Tables

**Table 1:** Characteristics of pediatric patients with acute lymphoblastic leukemia (ALL) enrolled in the Healthy Eating and Active Living (HEAL) pilot intervention.

Characteristics	N (%) or mean (SD) ^1^
Age, year, mean (SD)	6.1 (2.0)
Gender, N (%)	
Male	11 (73.3)
Female	4 (26.7)
Race/Ethnicity, N (%)	
Non-Hispanic Whites	9 (60.0)
Other	6 (40.0)
Time from diagnosis, months, mean (SD)	23.5 (8.25)
Treatment Status, N (%)	
On maintenance therapy	13 (86.7)
Post treatment completion (within two years)	2 (13.3)
Treatment Protocol	
AALL0932	6 (40.0)
AALL0434	1 (6.67)
DFCI11001	1 (6.67)
AALL1131	6 (40.0)
AALL1231	1 (6.67)
Cranial irradiation therapy, N (%)	
Yes	1 (6.67)
No	14 (93.3)
Prednisone equivalent dose, mg/m^2^, mean (SD) ^2^	5044 (2184)
BMI z-score, baseline, mean (SD) ^3^	0.9 (1.3)
BMI percentile, baseline, mean (SD)	70.4 (28.8)
Waist circumference, cm, baseline, mean (SD)	59.4 (6.1)

Abbreviations: ALL, acute lymphoblastic leukemia; BMI, body mass index; SD, standard deviation. ^1.^ N (%) were presented for categorical variables.Mean (SD) were presented for continuous variables. ^2.^ Cumulative dose of glucocorticoids is calculated by converting the dexamethasone does to a prednisone equivalent dose using 1 mg dexamethasone = 6.67 mg prednisone, and summing across the two medications. ^3.^ BMI z-score and percentile were calculated by comparing a child’s BMI to the mean BMI of children with same age and sex in the 2000 CDC growth chart.

**Table 2:** Changes in diet, physical activity, and weight status in pediatric patients of acute lymphoblastic leukemia (ALL), the Healthy Eating and Active Living (HEAL) pilot intervention.

Diet, Physical Activity, and Weight Status	Baseline	Post Intervention	Difference	P value ^1^
	Mean (SD)	Mean (SD)	Mean (95% CI)	
Food Groups				
Total fruits, servings/day	1.78 (1.67)	1.59 (1.19)	−0.19 (−1.19 to 0.81)	0.68
Whole fruits	1.03 (1.02)	0.81 (0.61)	0.22 (−0.27 to 0.70)	0.34
Fruit juices	0.97 (1.45)	0.15 (0.43)	−0.82 (−1.90 to 0.25)	0.12
Total vegetables, servings/day	0.86 (0.32)	0.93 (0.46)	0.07 (−0.20 to 0.33)	0.58
Dark-green vegetables	0.06 (0.09)	0.18 (0.29)	0.12 (−0.03 to 0.27)	0.11
Tomatoes	0.17 (0.14)	0.20 (0.19)	0.03 (−0.09 to 0.15)	0.61
Other red/orange vegetables	0.10 (0.10)	0.12 (0.21)	0.02 (−0.17 to 0.21)	0.80
Potatoes	**0.25 (0.19)**	**0.09 (0.16)**	**-0.16 (−0.30 to −0.03)**	**0.02**
Total grains, servings/day	7.36 (2.29)	7.98 (2.20)	0.62 (−0.68 to 1.92)	0.31
Whole grains	0.61 (0.65)	1.32 (1.72)	0.71 (−0.16 to 1.57)	0.10
Refined grains	6.75 (2.45)	6.67 (2.44)	−0.09 (−1.10 to 0.92)	0.85
Legumes, servings/day	0.03 (0.06)	0.03 (0.10)	0.005 (−0.06 to 0.07)	0.87
Nuts and seeds, servings/day	0.63 (1.17)	0.14 (0.36)	−0.49 (−1.26 to 0.29)	0.19
Total meats, oz. equiv./day	2.19 (1.70)	2.46 (2.23)	0.26 (−0.37 to 0.90)	0.38
Processed meats	0.98 (0.93)	0.92 (1.13)	−0.06 (−0.45 to 0.33)	0.74
Unprocessed red meats	0.46 (0.54)	0.54 (1.12)	0.08 (−0.48 to 0.63)	0.76
Poultry	0.69 (0.80)	0.84 (0.95)	0.15 (−0.25 to 0.56)	0.41
Fish and shellfish	0.07 (0.21)	0.15 (0.49)	0.09 (−0.30 to 0.48)	0.62
Total dairy products, servings/day	2.24 (1.15)	2.87 (1.40)	0.63 (−0.12 to 1.38)	0.09
Milk, servings/day	**1.38 (1.21)**	**1.92 (1.58)**	**0.54 (0.02 to 1.07)**	**0.04**
Cheese, servings/d	0.66 (0.32)	0.87 (0.68)	0.21 (−0.22 to 0.63)	0.30
Added sugars, g/day	**68.0 (29.4)**	**53.5 (26.4)**	**−14.6 (−29.5 to 0.4)**	**0.05**
Nutrients				
Total energy, kcal/day	1718 (384)	1600 (431)	−150 (−377 to 76.5)	0.17
Total fat, % E	32.2 (8.05)	33.7 (4.93)	1.53 (−2.31 to 5.37)	0.39
Saturated fat, % E	11.6 (2.60)	12.5 (2.01)	0.88 (−0.43 to 2.20)	0.16
Monounsaturated fat, % E	11.8 (3.87)	11.8 (2.57)	0.08 (−1.45 to 1.62)	0.91
Polyunsaturated fat, % E	6.13 (2.13)	6.31 (1.26)	0.17 (−1.45 to 1.80)	0.81
Protein, % E	**12.4 (2.47)**	**15.0 (2.16)**	**2.54 (0.22 to 4.87)**	**0.04**
Carbohydrate, % E	56.9 (9.19)	52.4 (6.72)	−4.52 (−9.16 to 0.11)	0.05
Fiber, g/day	14.9 (5.27)	15.7 (5.45)	0.79 (−1.26 to 2.86)	0.40
Sodium, mg/day	3314 (619)	3513 (454)	199 (−254 to 653)	0.35
Cholesterol, mg/day	173.4 (94.2)	218.2 (110)	44.8 (−45.5 to 135)	0.29
Potassium, mg/day	2243 (491)	2283 (381)	40.2 (−206 to 286)	0.72
Calcium, mg/day	**1070 (377)**	**1372 (337)**	**302 (25.1 to 579)**	**0.04**
Vitamin D, mcg/day	5.67 (3.21)	8.20 (4.99)	2.53 (−0.13 to 5.19)	0.06
Healthy Eating Index-2015	44.5 (9.08)	47.7 (7.91)	3.14 (−3.55 to 9.82)	0.29
Physical activity, minutes/day				
Light	231 (112)	155.8 (64.1)	−75.1 (−156 to 7.64)	0.07
Moderate	23.6 (13.1)	16.2 (8.02)	−7.42 (−17.7 to 2.87)	0.14
Vigorous	4.27 (3.41)	4.03 (2.73)	−0.23 (−2.82 to 2.36)	0.85
Moderate-to-vigorous	27.9 (16.3)	20.2 (10.0)	−7.65 (−20.1 to 4.76)	0.20
Anthropometrics				
BMI z-score	0.79 (1.14)	0.80 (1.26)	0.02 (−0.38 to 0.41)	0.93
BMI percentile	70.3 (28.8)	71.6 (31.7)	1.31 (−10.6 to 13.3)	0.81
Waist circumference, cm	59.5 (6.34)	60.4 (8.0)	0.86 (−1.96 to 3.68)	0.52

Abbreviations: ALL, acute lymphoblastic leukemia; SD, standard deviation; % E, percent of energy. ^1^ Significant differences before and after the intervention were bolded.

**Table 3:** Changes in parenting style and practices in nutrition and physical activity in parents of pediatric patients of acute lymphoblastic leukemia (ALL), the Healthy Eating and Active Living (HEAL) pilot intervention.

Parenting Styles and Practices	Baseline	Post Intervention	Difference	P value ^3^
	Mean (SD)	Mean (SD)	Mean (95% CI)	
Feeding practices ^1^				
Pressure to eat	2.69 (1.22)	2.10 (0.70)	**−0.60 (−1.12 to −0.07)**	**0.03**
Monitoring	3.72 (0.83)	3.69 (0.92)	−0.03 (−0.52 to 0.47)	0.91
Restriction	3.31 (0.65)	3.11 (0.79)	−0.20 (−0.63 to 0.23)	0.33
Physical activity practices ^2^				
Logistic support	3.00 (0.82)	3.21 (0.63)	0.21 (−0.11 to 0.52)	0.18
Explicit modeling	3.13 (0.76)	3.14 (0.84)	0.02 (−0.34 to 0.38)	0.91
Limit setting	4.15 (0.93)	4.29 (1.09)	0.13 (−0.51 to 0.78)	0.66
Parenting style	N (%)	N (%)		
Authoritative	3 (23.08)	3 (23.08)	---	0.62
Authoritarian	5 (38.46)	5 (38.46)	---	
Indulgent	4 (30.77)	2 (15.38)	---	
Uninvolved	1 (7.69)	3 (23.08)	---	

^1.^ Feeding practices were assessed using the Child Feeding Questionnaire (CFQ) that measures three feeding practice subscales (restriction, monitoring, and pressure to eat). ^2.^ Physical activity practices were assessed using items that measures logistical support, explicit modeling, and limit setting in relation to child’s physical activity. Parenting styles were assessed using the Parenting Dimensions Inventory Short Version (PDI-S). Five parenting dimensions were categorized into four parenting styles (authoritative, authoritarian, indulgent, and uninvolved). ^3.^ Significant differences before and after the intervention were bolded.
